# Evidence for Asymmetrical Divergence-Gene Flow of Nuclear Loci, but Not Mitochondrial Loci, between Seabird Sister Species: Blue-Footed (*Sula nebouxii*) and Peruvian (*S. variegata*) Boobies

**DOI:** 10.1371/journal.pone.0062256

**Published:** 2013-04-17

**Authors:** Scott A. Taylor, David J. Anderson, Vicki L. Friesen

**Affiliations:** 1 Queen's University, Department of Biology, Kingston, Canada; 2 Wake Forest University, Department of Biology, Winston-Salem, North Carolina, United States of America; Tuscia University, Italy

## Abstract

Understanding the process of speciation requires understanding how gene flow influences divergence. Recent analyses indicate that divergence can take place despite gene flow and that the sex chromosomes can exhibit different levels of gene flow than autosomes and mitochondrial DNA. Using an eight marker dataset including autosomal, z-linked, and mitochondrial loci we tested the hypothesis that blue-footed (*Sula nebouxii*) and Peruvian (*S. variegata*) boobies diverged from their common ancestor with gene flow, paying specific attention to the differences in gene flow estimates from nuclear and mitochondrial markers. We found no gene flow at mitochondrial markers, but found evidence from the combined autosomal and z-linked dataset that blue-footed and Peruvian boobies experienced asymmetrical gene flow during or after their initial divergence, predominantly from Peruvian boobies into blue-footed boobies. This gene exchange may have occurred either sporadically between periods of allopatry, or regularly throughout the divergence process. Our results add to growing evidence that diverging species can remain distinct but exchange genes.

## Introduction

Understanding speciation necessarily involves understanding the influence of gene flow on the process of divergence [1]–[4]. Until recently, quantifying gene flow and introgression during and following divergence was difficult; however, advances both in sequencing technology and in analytical methods based on coalescent theory are removing these barriers [5].

The predominant historical and contemporary view of speciation invokes divergence in allopatry with a permanent interruption in gene flow between diverging populations [6]. However, the allopatric model has failed to provide satisfactory explanations for some evolutionary events [2], [7]–[10]. New analytical methods based on coalescent theory have revealed that divergence occurs with some level of gene flow more commonly than originally thought [4], [5], but estimating the timing of gene flow events remains difficult [11]–[13]. Some studies have even tested the likelihood of one divergence scenario over another statistically, allowing conclusions about divergence and speciation to be expressed with an explicit level of confidence [10], [14]–[16]. To evaluate the frequency of divergence with versus without gene flow, the two scenarios must be tested explicitly in a diverse array of taxa.

New analytical methods also facilitate the investigation of hybridization and introgression between closely related taxa [14], [16], [17]. Until recently, introgression has been evaluated almost exclusively using cline theory to examine allele and haplotype frequency changes across hybrid zones [18]–[20]. These studies often report reduced introgression of some loci compared to others, in particular reduced introgression of the mitochondrial genome and, when examined, of sex-linked loci [19]. However, cline theory has limitations: differences in effective population size can influence cline width and are not taken into consideration by cline-based analyses, and most cline-based analyses involve secondary contact zones rather than situations of divergence with gene flow (i.e. primary contact zones) [14].

Results from studies of population divergence, hybridization, and speciation indicate that the sex chromosomes can exhibit different levels of gene flow than autosomes and mitochondrial DNA [4], [14], [15], [21]–[26]. Birds have a zz/zw sex chromosome system where the possession of the w chromosome makes an individual female [27]. Like the mitochondria, the w chromosome is inherited by female offspring from their mother; the z chromosome is inherited by female offspring from their father. Sons inherit a z chromosome from each parent [27].

### Study system

**Figure 1:**
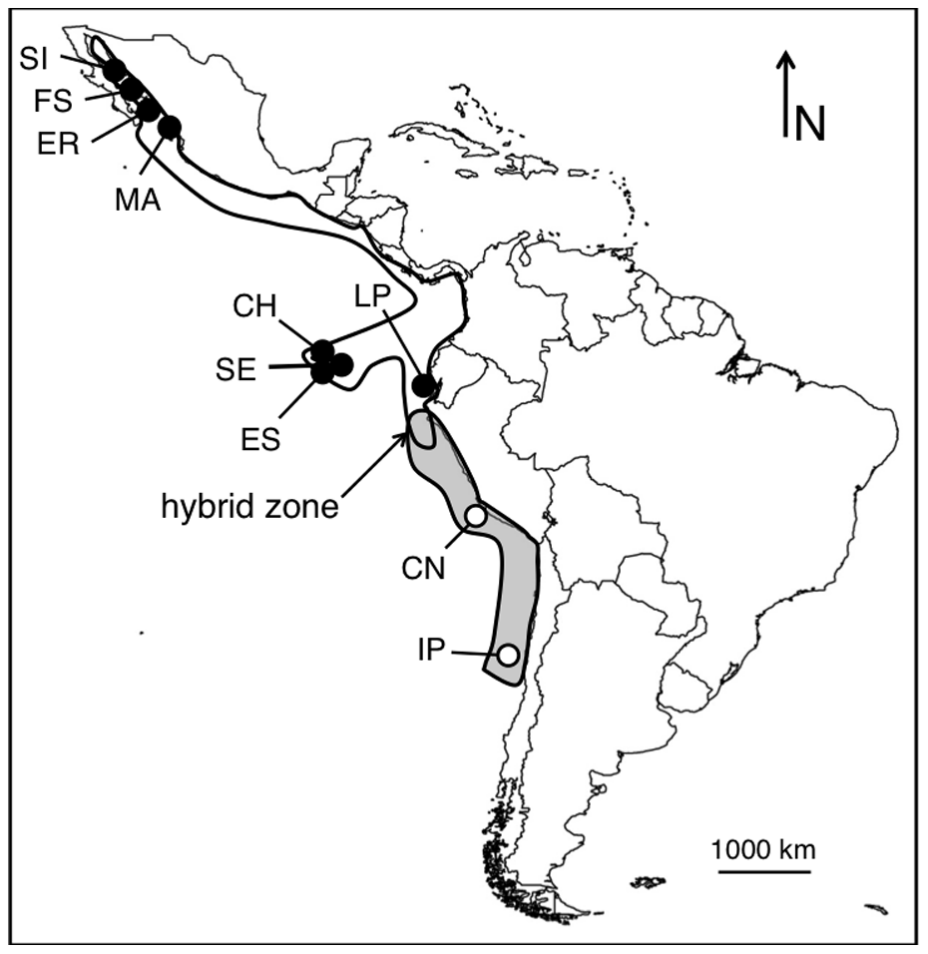
Map of Central and South America showing sampling locations. Approximate breeding distributions in white for blue-footed boobies and grey for Peruvian boobies; blue-footed booby sample sites indicated by black circles, Peruvian booby sample sites indicated by white circles. Isla San Ildefonso, Mexico (SI; 5), Farallon de San Ignacio, Mexico (FS; 5), El Rancho, Mexico (ER; 4), Islas Marietas, Mexico (MA; 1), La Plata, Ecuador (LP; 4), Champion Island, Galapagos (CH; 1), Seymour Island, Galapagos (SE; 1), Espanola Island, Galapagos (ES; 1), Isla Chincha Norte, Peru (CN; 6), Isla Pajaros, Chile (IP; 10). Numbers in parentheses are number of individuals sampled per colony.

Blue-footed and Peruvian boobies are large, plunge diving, primarily tropical members of the Sulidae (Aves: Pelecaniformes) found throughout the eastern tropical Pacific Ocean (Fig. 1). They exhibit weak (blue-footed boobies) [28] or no (Peruvian boobies) [29] intraspecific population structure at neutral loci. They are sister species that diverged recently (0.25–1.1 mya) [30], [31], and that hybridize occasionally where their ranges overlap on two islands in northern Peru, Isla Lobos de Tierra and Isla Lobos de Afuera (Fig. 1) [30]–[35]. On Lobos de Tierra, blue-footed boobies outnumber Peruvian boobies by an order of magnitude and the species tend to form distinct colonies. On Lobos de Afuera, blue-footed and Peruvian boobies exist at relatively equal numbers, and overlapping breeding aggregations on this island are not uncommon (Taylor pers obs). Interspecific courting behaviour has been documented on both islands [32], [33], [35]; however, the level of hybridization that occurs in this system does not violate the modified biological species concept [36]. Both species have restricted distributions within the eastern Tropical Pacific, but have relatively large and stable populations [37]. The species are highly divergent at the mitochondrial control region and are significantly differentiated at multiple unlinked nuclear loci [28], [29], [35].

These species are also well differentiated morphologically [34], and a number of these differences may be the product of sexual selection [38]. Blue-footed boobies possess bright blue feet and yellow irides, while Peruvian boobies possess grey feet and have dark red irides. Foot colouration is known to be important for sexual selection and signalling in blue-footed boobies [39], and both the diversity of foot colour within members of the Sulidae and evidence that bare part colouration is important in signalling in seabirds [38] suggest that foot colour is a target of sexual selection in these species.

Contemporary hybridization between blue-footed and Peruvian boobies tends to occur between female Peruvian boobies and male blue-footed boobies, and produces viable F1, predominantly male, individuals [32], [34], [35]; however, introgression across the hybrid zone is low, suggesting that backcross hybrids experience strong negative selection [35]. A genetic survey of 321 individuals across the range of each species revealed five F1 hybrids (four male and one female), two likely backcross hybrids, and no evidence of mitochondrial introgression, potentially due to strong premating isolation and strong selection against hybrids [34], [35].

Although blue-footed and Peruvian boobies are well differentiated genetically and morphologically, previous research suggested that they may not have diverged from their common ancestor in a classic allopatric manner, in the absence of gene flow. Given the lack of vicariance events since their divergence and their parapatric breeding distribution, Friesen & Anderson (1997) suggested that the most likely mode of speciation for this species pair was through parapatric divergence. Divergence in parapatry involves selection that reduces gene flow, inducing reproductive isolation. Indeed, the species possess the geographic signature of parapatric speciation: they are young sister species that experience limited hybridization where their ranges overlap at an ecotone, and no physical barriers to dispersal exist between their ranges.

The nature of the blue-footed/Peruvian booby hybrid zone is unknown, but the possibility that it is a primary contact zone cannot be ruled out. Fossil evidence indicates that sulids have occupied the eastern Tropical Pacific, including the Peruvian upwelling and islands within the North Equatorial Counter Current, since the middle Miocene [40]. The breeding islands shared by blue-footed and Peruvian boobies have been occupied by both species at least since the beginning of recorded history, and probably for thousands of years prior to their discovery [41]. Additionally, blue-footed and Peruvian boobies diverged from their common ancestor long after the establishment of the Peruvian upwelling, to which Peruvian boobies are endemic and highly specialized [30], [31], [37], [42], and both species regularly travel long distances within the eastern tropical Pacific [43], [44], which could increase encounters with heterospecifics and potentially facilitate interspecific gene flow. Given these facts, the contact zone may have existed regularly, but potentially between periods of allopatry, throughout the evolutionary history of these birds and we hypothesize that blue-footed and Peruvian boobies diverged from their common ancestor via a mechanism other than the strict allopatric model.

### Objectives

The primary objective of this paper is to evaluate the hypothesis that the divergence of blue-footed and Peruvian boobies from their common ancestor did not follow a strict allopatric model. We evaluate this hypothesis using Bayesian and coalescent theory to compare models of divergence with gene flow to those where gene flow is constrained to zero. Second, we compare estimates of gene flow during divergence between different marker types (autosomal, sex-linked, and mitochondrial). Note that the method employed tests for gene flow during divergence, not contemporary gene flow, and accounts for recent shared ancestry [45].

## Methods

### Ethics statement

Sampling methods were approved by Queen's University Animal Care Committee (permit: Friesen-2007-010-Or), in compliance with all national and international guidelines.

### Sample collection and laboratory methods

Although hybridization has been recorded between blue-footed and Peruvian boobies it is restricted to two islands in northern Peru and does not cause extensive introgression [34], [35] However, to minimize the influence of ongoing hybridization on gene flow and divergence time estimates, samples for each species were assembled from areas of allopatry. Sampling included a total of 21 blue-footed boobies from Mexico, Galapagos, and Ecuador, and 15 Peruvian boobies from southern Peru and central Chile (Fig. 1). The sample sizes are on par with similar studies in birds [14], [15]. Samples consisted of blood collected and stored as described in Taylor *et al*. (2010, 2011a,b). DNA was extracted either using a PureLink® extraction kit (Invitrogen: Burlington, Canada) or a standard protease-K phenol/chloroform technique [46].

#### Introns

Initially, up to 10 birds of each species were screened for sequence variation at 12 autosomal and seven z-linked introns [47], [48]. Sequences of nine of 19 introns were identical in blue-footed and Peruvian boobies, while the other introns showed low levels of variation either within or between species (Table S1). Seven introns, including introns in the genes for α-enolase (ENOL) [47], lipoprotein lipase [LIPO; V.L.F unpubl. data], triosephosphate isomerase (TIM; V.L.F unpulb. Data), β-fibrinogen (FIB) [49] and three from the z chromosome (24105, 25189, ALDOB) [48] were chosen for further analyses based on amplification success and variability (Table 1). Given the results (see Results) we do not believe intron selection introduced any systematic biases in the data.

**Table 1:** Details of loci used to evaluate divergence of blue-footed and Peruvian boobies, including sequence length, number of chromosomes (autosomal loci) or individuals (mtDNA) analysed (N), number of variable sites (*V*) haplotype diversity (*h*), nucleotide diversity (π), and estimates of Tajima's D.

Locus	Species	Length (bp)	N	*V*	*h*	π	Tajima's D
mtDNA							
ND2+CYTB	Blue-footed	1184	12	118	0.97	0.0049	−0.009
	Peruvian		10		1.00	0.0072	−0.79
Autosomal							
FIB	Blue-footed	506	24	1	0.00	0.000	-
	Peruvian		22		0.09	0.0002	−1.2
ENOL	Blue-footed	165	42	4	0.05	0.0003	−1.1
	Peruvian		30		0.07	0.0004	−1.1
TIM	Blue-footed	211	24	2	0.16	0.0008	−0.68
	Peruvian		22		0.40	0.0021	−1.2
LIPO	Blue-footed	114	24	1	0.51	0.0044	1.5
	Peruvian		22		0.00	0.000	-
Sex-linked							
24105	Blue-footed	452	28	2	0.36	0.0009	−0.38
	Peruvian		21		0.00	0.000	-
ALDOB	Blue-footed	428	28	3	0.35	0.0008	0.57
	Peruvian		24		0.49	0.0011	1.4
25189	Blue-footed	470	16	0	0.00	0.000	-
	Peruvian		17		0.00	0.000	-

Polymerase chain reactions (PCR) were performed in 25 µL volumes containing 1× Multiplex Mix (QIAGEN, Mississauga, Ontario), 0.4 mM each of forward and reverse primers, and ∼5 ng of DNA template. PCR products were sequenced at Genome Quebec (McGill University, Quebec) using a 3730xl DNA Analyzer system (Applied Biosystems: Streetsville, Ontario). Sequences were aligned using CULSTALW [50] as implemented in BioEdit Ver. 7.0.5.3 [51], and each variable site was confirmed against the sequencing trace using FinchTV Ver. 1.4.0 (Geospiza Inc., www.geospiza.com/finchtv.html).

To ensure that females were not scored as homozygous males for z-chromosome intron sequences, and that the appropriate numbers of alleles were included in coalescent analyses, all individuals were sexed using the universal avian sexing primers 2550F and 2718R [25]. PCR products were subjected to electrophoresis and visualized on 2% agarose gels.

Allele sequences were inferred directly from the sequencing trace for homozygous individuals, for heterozygous individuals that possessed a single variable site, and for all females at z-linked introns. When possible, sequences of homozygotes were used to infer allele sequences for heterozygotes. The program PHASE (Version 2.1) [52], [53] was used to determine the alleles possessed by heterozygous individuals when a homozygous individual for an allele was not present, and for males heterozygous at sex-linked loci. Appropriate run length was determined by examining the consistency of the haplotype frequency estimates across ten runs. All PHASE analyses were run with a burn-in of 1000 iterations, 2000 iterations after burn-in, and the default thinning interval of one. Phase determination was conservatively accepted at probabilities of 0.85 and higher: haplotypes inferred by PHASE with a probability greater than 0.60 are likely to reflect haplotypes found during cloning [54]. Individuals for which the program could not determine the allele phase with this level of certainty were removed from subsequent analyses.

#### Mitochondrial markers

A 721 bp fragment of the Cytochrome *b* gene (Cyt *b*) and a 463 bp fragment of the ND2 gene were amplified and sequenced for 12 blue-footed boobies and ten Peruvian boobies as for introns above [47], [55]. Fewer individuals were used for the mitochondrial analyses given the likelihood that the markers would be reciprocally monophyletic, an expectation from previous work [34], [35].

#### Data analyses

Each intron was examined for intralocus recombination using the four-gamete test as implemented in DnaSP ver. 4.10 [56], and only the largest non-recombining sequence block of the intron was used for subsequent analyses. DnaSP was also used to calculate Tajima's D to determine if variation in any locus deviated from neutral expectations within either species [57], and to calculate haplotype diversity (*h*) and nucleotide diversity (π). All pairs of intron loci were tested for deviations from linkage equilibrium using ARLEQUIN [58]. Mutational relationships among intron alleles were inferred by constructing statistical parsimony networks in TCS Ver. 1.21 [59].

The program IMa [45] was used to contrast models of divergence with gene flow versus strictly allopatric differentiation. IMa implements the coalescent based “Isolation-with-Migration analytic” model and makes a number of assumptions about the data, including that 1) the sampled populations are each other's closest relatives and no unsampled populations are exchanging genes with them, 2) the loci being used are selectively neutral, 3) there is no within-locus recombination, 4) there is free between-locus recombination, and 5) the correct mutation model is chosen for the data. To the best of our knowledge, the data analysed here fit the assumptions of the Isolation-with-Migration model. Blue-footed and Peruvian boobies are sister species [30], [31] and exhibit little (blue-footed booby) [28] or no (Peruvian booby) [29] population differentiation across their geographic ranges (assumption 1). The loci used here appear selectively neutral (Table 1; assumption 2), we chose the largest non-recombining blocks of sequence at each locus (see above: assumption 3), the loci are not linked (see below; assumption 4), and the appropriate mutation model was selected (infinite sites model or HKY model) based on examination of the variable sites in each intron. For the present analyses IMa was first used to estimate six demographic parameters including divergence time (*t*), gene flow (*m_1_*–gene flow from population two to population one going forwards in time, and *m_2_*–gene flow from population one to population two going forward in time), and effective population size (*q_1_*–effective size of population one at time of splitting, *q_2_*–effective size of population two at splitting, and *q_A_*–effective size of the ancestral population) [45], which constitutes the full IMa model. Gene flow estimates were considered significantly greater than zero if the lower bin of the 90% HPD did not include zero.

To evaluate the relative levels of gene flow at autosomal, sex-linked, and mitochondrial markers since the divergence of blue-footed and Peruvian boobies, four datasets were analysed using IMa, each of which included all available individuals. The datasets in the full model analyses included (1) autosomal and z-linked loci, (2) autosomal loci, (3) z-linked loci, and (4) mitochondrial loci. IMa can accommodate data with different patterns of inheritance by assigning the appropriate scalar values, which we implemented here. Dataset 4 included only mitochondrial loci, and was analyzed using the full model and a simplified model where gene flow was constrained to zero because the mitochondrial data were reciprocally monophyletic (see Results). The mitochondrial loci were not added to the combined autosomal and z-linked data set, acknowledging the potential for different migration rates across the loci due to differences in dispersal tendencies between male and female sulids [60], [61]. We acknowledge that our rationale for not including mtDNA in a full analysis could also be applied to z-linked markers to a lesser extent. We did not carry out a full analysis using mtDNA because we did not feel it would add clarity to the overall results.

Initial runs of IMa were given wide priors (*q_1_*, *q_2_*, *q_A_* = 10; *m_1_*, *m_2_* = 60, *t* = 5), which were used to determine priors that allowed each parameter estimate to reach convergence. After appropriate priors were identified each dataset was run three times with identical priors and a geometric heating scheme (g1 = 0.96, g2 = 0.90) that utilized at least 30 chains, with a burn-in of 10^6^ steps and an additional run length of at least 10^7^ steps. Each of these three runs was started using a different random number seed. To evaluate chain mixing and parameter convergence, trend line plots were visually inspected and effective sample size (ESS) values were monitored. ESS values for all parameters were allowed to reach at least 100 for all final runs.

Following run convergence, the “Load-trees” (L) mode of IMa was used to test the fit of dataset 1 (autosomal + z linked loci) to the simplified model where gene flow (*m_1_*, *m_2_*) was constrained to zero. The 90% highest posterior density (HPD) intervals for the migration parameters estimated from datasets 2 and 3 all included the lowest bin of the probability distribution (see results: Table 2). As such, we did not feel it was appropriate to test the fit of these datasets to the simplified model. This simplified model approximates an allopatric divergence scenario, the likelihood of which was then compared statistically to a scenario of divergence with gene flow (the full model). The fit of the data to each divergence scenario was evaluated using a nested model approach as implemented in L mode with at least 10^5^ trees [45]. The nested model approach calculates log-likelihood ratio statistics for all possible nested models [45]. The significance of these log-likelihood ratio statistics can then be evaluated using a chi-square test [45], providing a quantitative inference of the consistency of the data with an allopatric divergence scenario versus an alternative scenario of divergence-with-gene-flow.

**Table 2:** Demographic parameter estimates with 90% highest posterior density intervals for divergence of blue-footed and Peruvian boobies.

		Blue-footed	Peruvian	ancestral	Peruvian blue-footed	blue-footed Peruvian
Dataset	t (years)	N_1_	N_2_	N_A_	2N_1_m_1_	2N_2_m_2_
**1**(Autosomal + z-linked loci)	1,800,000	6500(1800–32,000)	12,000(2400–49,000)	200(200–380,000)	0.14(0.02–0.64)	0.013(0–0.20)
**2**(Autosomal loci)	180,000	10,000(2200–38,000)	35,000(8000–300,000)	360(100–180,000)	0.10(0–0.34)	0.12(0–0.92)
**3**(Z-linked loci)	2,800,000	30,000(1600–180,000)	7,000(650–65,000)	490(650–850,000)	0.28(0–0.95)	0.00(0–0.09)
**4**(Mitochondrial)	2,000,000	42,000(2500–240,000)	295,000(100,000–756,000)	469(469–220,000)	0.00(0–2)	0.00(0–2)

t  =  years since divergence from common ancestor, N  =  effective population size, 2Nm  =  per generation population migration rate.

Because migration estimates indicated unidirectional gene flow (see results), we also estimated the likelihood of a model where gene flow from blue-footed boobies to Peruvian boobies (*m_2_*) was restricted to zero, but gene flow from Peruvian boobies to blue-footed boobies (*m_1_*) was allowed. The fit of the data to the unidirectional gene flow model was then compared to the full model as described above, again only for dataset 1.

All IMa parameter estimates are scaled to the neutral mutation rate (μ). To convert divergence time (*t*) to years and effective population sizes (*q_1_*, *q_2_*, and *q_A_*) to individuals, intron mutation rates (substitutions/locus/year) were calculated using the methods described in Peters et al. 2008 [62]. An estimate of the phalacrocoracid/sulid divergence time was obtained using a relaxed molecular clock and several fossil calibrations (35 million years) [31], [62]. We acknowledge that substitution rates for these recently diverged species may be higher than estimated using the phalacrocoracid/sulid divergence [63]. Using the aforementioned laboratory protocols, sequence was obtained for the ENOL, 24105, 25189, LIPO, TIM and ALDOB introns from one red-faced cormorant (*Phalacrocorax urile*) and up to five sulid species in addition to blue-footed and Peruvian boobies. BEAST Version 1.5.4 [64] was then used to estimate the node heights between the root (phalacrocoracid/sulid) and tips (contemporary cormorants and boobies) for each locus. This method samples over a range of probable trees based on the data to account for stochastic variation in genetic processes [64]. These values were subsequently divided by 35 million years to yield substitutions/site/year for each locus, and then multiplied by the length of each locus to determine the mutation rate in substitutions/locus/year (Table S1). The estimated mutation rate for FIB was taken from [65], where a similar approach was implemented.

Estimates of demographic parameters were made using the geometric mean of the appropriate mutation rates calculated from intron loci: dataset 1, 2.65×10^−7^; dataset 2, 3.31×10^−7^; dataset 3, 1.91×10^−7^; dataset 4, 1.9×10^−6^. [45]. Mutation rates differ between loci; however, IMa can accommodate these differences and all geometric mean mutation rates were calculated appropriately. We multiplied the average intron mutation rate by ten to generate an approximate mutation rate for the mitochondrial locus [14], [66]. Conversion of IMa estimates to demographic parameters followed methods described in IMa documentation: per year mutation rates were converted to per generation mutation rates using a four year generation time [61]. Because robust estimates of divergence times between blue-footed and Peruvian boobies have been calculated from both mitochondrial [30] and nuclear [31] data, divergence time estimates were not of primary concern for the present study.

Recent simulation studies have shown that there are a number of limitations to the IM approach, but have also shown that the model is robust to violations of a number of assumptions [12]. The presence of population structure and recombination within loci, for example, do not appear to have a large impact on parameter estimates; however, ancestral population structure, variation in gene flow through time, and deviations from the assigned mutation models can significantly impact parameter estimates [12].

Phylogenetic analysis of mitochondrial DNA (cytb and ND2) was conducted using MrBayes 3.1 [67], [68]. MrModelTest ver 2.3 [69] was used to determine the appropriate substitution model, the parameters of which were allowed to vary during MrBayes analyses. We performed two runs for 10,000,000 generations each and the Markov chains were sampled every 1000 generations. To test for convergence we compared posterior probabilities of clades from independent runs using the program Are We There Yet [70].

## Results

**Figure 2:**
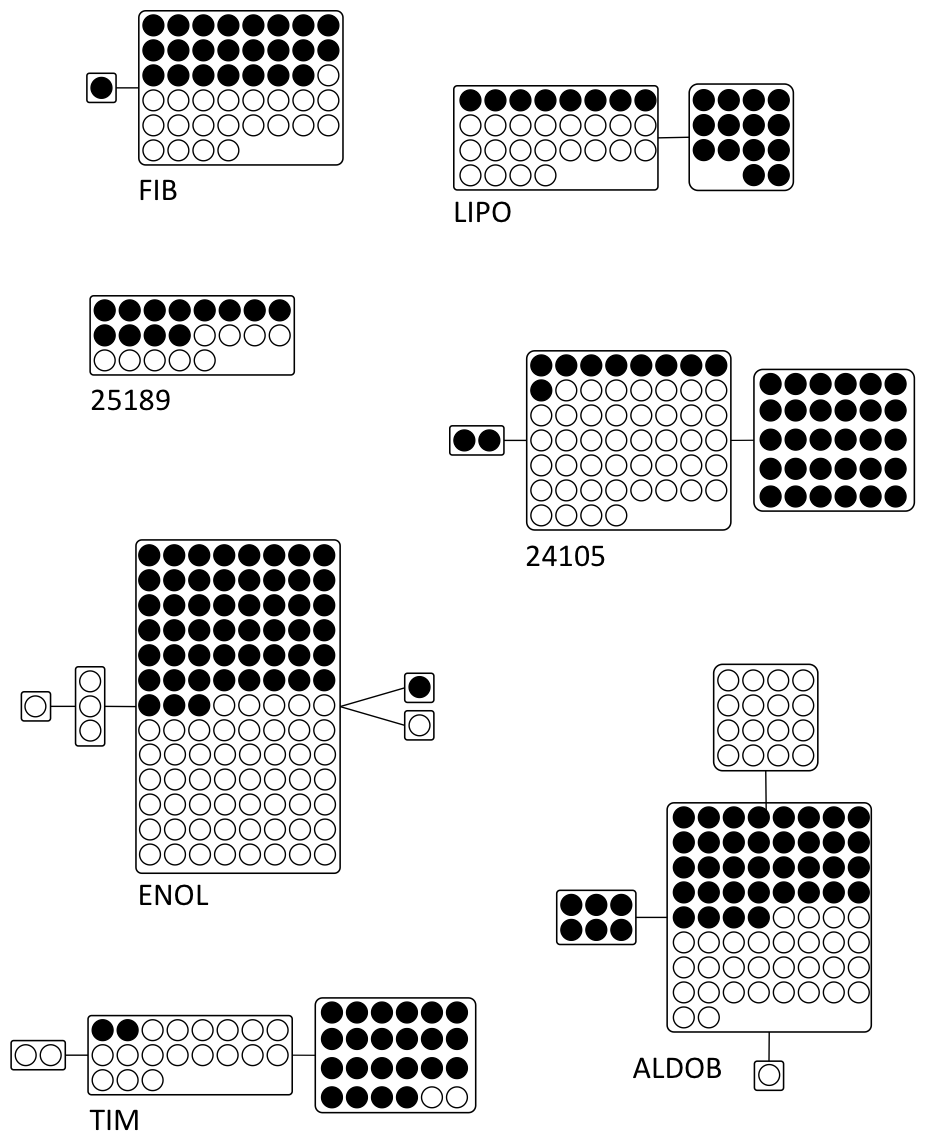
Statistical parsimony networks of haplotypes from autosomal and z-linked introns. Boxes represent haplotypes, black and white circles represent sequences found in blue-footed boobies or Peruvian boobies, respectively. Intron abbreviations as in Table 1.

The average length of non-recombining sequence blocks for the nuclear markers was 335 bp. Lengths of sequence blocks ranged from 114 bp for LIPO to 1184 for the concatenated mitochondrial sequence of Cyt *b* and ND2 (Table 1). Haplotype and nucleotide diversity were variable, ranging from 0.00 to 1.00 and from 0.00 to 0.0072 respectively (Table 1). PHASE was required to determine only ENOL haplotypes; however, reliable sequences were not obtained for all individuals at all loci (Table 1). No loci showed deviations from neutrality using Tajima's D (all P>0.05; Table 1), or from linkage equilibrium. Shared alleles existed for all introns (Fig. 2); however, blue-footed and Peruvian boobies were reciprocally monophyletic at mitochondrial loci, a pattern also reported in Taylor et al. (2010, 2012). Intron and mitochondrial sequences greater than 200 bases have been deposited in GenBank (Accession # KC577244-KC577266). Sequences shorter than 200 bases have been included in Appendix S1.

**Figure 3:**
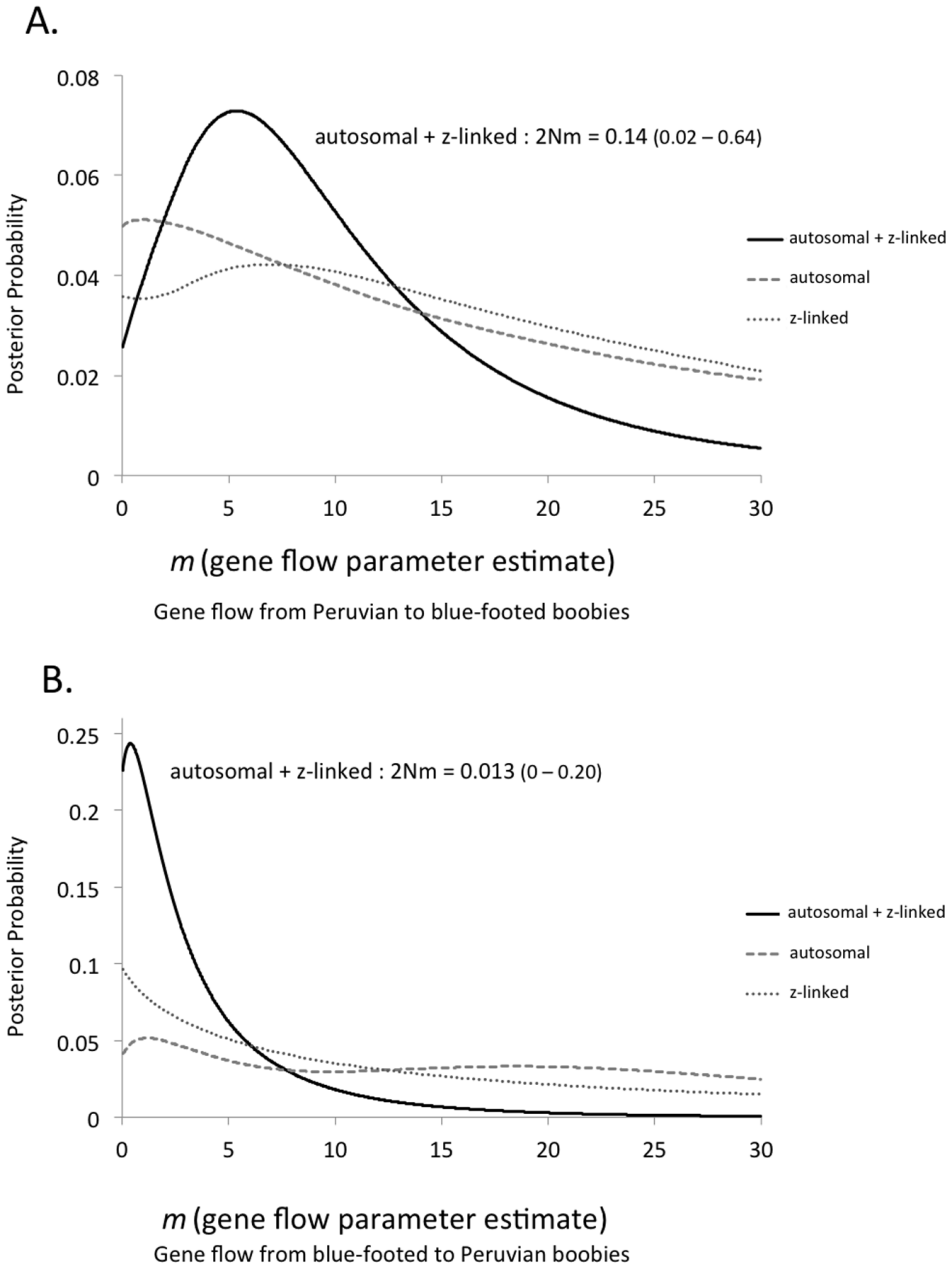
Posterior probability distributions from IMa [**45**] for gene flow between blue-footed and Peruvian boobies for the total, autosomal, and z-linked datasets. Gene flow parameters *m_1_* and *m_2_* scaled by the mutation rate. 2Nm  =  population migration rate per generation. A) Gene flow from Peruvian boobies to blue-footed boobies (*m_1_*). B) Gene flow from blue-footed boobies to Peruvian boobies (*m_2_*).

IMa had difficulty reaching convergence when datasets 2 (autosomal) and 3 (z-linked) were analysed individually, suggesting that the loci in these datasets did not, on their own, contain enough information for the full six-parameter model. That being said, estimates of migration from z-linked loci differed from the autosomal and mitochondrial estimates. Due to a lack of convergence, we focus our discussion on datasets 1 (autosomal + z-linked) and 4 (mitochondrial), for which IMa did not have difficulty reaching convergence. We have, however, included demographic parameters estimated from all datasets in Table 2,

The 90% highest posterior density (HPD) intervals of estimated effective population size for blue-footed boobies and Peruvian boobies overlapped broadly for each dataset. Estimates of divergence time were of similar magnitude to previous estimates [30], [31], and similar between datasets 1 and 4 (Table 2). Estimates of gene flow from Peruvian boobies into blue-footed boobies (*m_1_*) were significantly greater than zero for dataset 1 (Fig. 3A). The estimate of gene flow from blue-footed boobies to Peruvian boobies (*m_2_*) was not significantly different from zero for any dataset, but the point estimate was greater than zero for dataset 2 (Table 2; Fig. 3B). Estimates of gene flow from dataset 4 (mitochondrial loci) were not significantly different than zero (Table 2).

The strict allopatric model of divergence was rejected for dataset 1, supporting a divergence with gene flow scenario (Table 3). The unidirectional gene flow model, which included only gene flow from Peruvian boobies to blue-footed boobies, could not be rejected for dataset 1, indicating that gene flow during divergence may have been asymmetrical, in agreement with reported estimates of gene flow (Table 2, Table 3).

**Table 3:** Results from nested model testing as implemented in “Load-trees” mode of IMa for the divergence of blue-footed and Peruvian boobies.

Test	Dataset	log(p'(Θ|X))	d.f.	2LLR	P
***Gene flow vs. strict allopatry***					
1	Autosomal + z-linked loci	0.25	2	6.15	**<0.05**
***Gene flow vs. unidirectional gene flow***					
2	Autosomal + z-linked loci	3.23	2	0.18	>0.05

Test one was between the full six parameter model of divergence (θ_1_, θ_2_, θ_A_, *m_1_*, *m_2_*, *t*) and the strict allopatric model of divergence (θ_1_, θ_2_, θ_A_, *m_1_* = 0, *m_2_* = 0, *t*). Test two was between the full, six parameter model of divergence and a model of divergence without gene flow from blue-footed boobies into Peruvian boobies (θ_1_, θ_2_, θ_A_, *m_1_*, *m_2_* = 0, *t*). Critical χ^2^ value = 5.99. Significance (P<0.05) indicates that the reduced model can be rejected in favour of the model of divergence with gene flow.

## Discussion

A strict allopatric model of divergence was rejected by the present results: the simplest model that fit the data included unidirectional gene flow from Peruvian into blue-footed boobies. IMa estimates of gene flow from Peruvian boobies to blue-footed boobies were significantly greater than zero for dataset 1 (autosomal + z-linked loci), while estimates of gene flow from blue-footed to Peruvian boobies were not significantly greater than zero for any dataset. Thus, blue-footed and Peruvian boobies appear to have experienced asymmetric gene flow during their speciation history, potentially the result of alternation between allopatry and parapatry [4]. Recent work has highlighted that strict allopatric models of divergence are not adequate to explain the divergence of numerous taxa [5], [8], [71], [72]. Additionally, divergence of mitochondrial genes was higher than nuclear loci and no gene flow was detected for dataset 4 (mitochondrial).

### High divergence of mitochondrial genes

Divergence between blue-footed and Peruvian boobies was lower at nuclear loci compared to mitochondrial loci, and mitochondrial loci did not show evidence of gene flow between the species (Table 2), a finding congruent with the data presented in Taylor et al. (2012). Additionally, the divergence time estimated from mitochondrial loci was earlier than the divergence time estimated by Friesen & Anderson (1997) (0.2 million years ago), but of the same magnitude as estimates in Patterson et al. (2010) (1.1 million years ago). The present study included ND2 in the concatenated mitochondrial sequence, which may have inflated the divergence time estimate compared to Friesen and Anderson (1997) (Table 2).

The absence of introgression at mitochondrial loci and the high level of divergence at mitochondrial loci found in the present study and in Taylor et al. (2012) could be the result of the effect described by Haldane's Rule, which states that the heterogametic sex should suffer a greater fitness disadvantage (e.g., is more likely to be sterile) as an F1 hybrid; however, female F1 hybrids appear to be fertile [34]. In this case Haldane's Rule may operate as in other z-chromosome systems such as butterflies, where hybrid breakdown can occur in later generation hybrids [73]. Whether backcross hybrids suffer hybrid breakdown due to mito-nuclear dysfunction or they experience environmental selection is unknown. Given that numerous subunits of the electron transport chain are encoded in nuclear DNA, mito-nuclear dysfunction may play a role in reduced hybrid fitness. The mitochondria and related nuclear genes encoding electron transport chain subunits could be under strong divergent natural selection [74]. Blue-footed and Peruvian boobies inhabit different marine environments throughout much of their ranges, and studies on the two islands where both species breed indicate that they have different environmental tolerances [75], [76]. The importance of the mitochondria for general physiological function is well established and comparative studies of physiology of these species may suggest sources of selection during divergence.

### Asymmetrical gene flow

**Figure 4:**
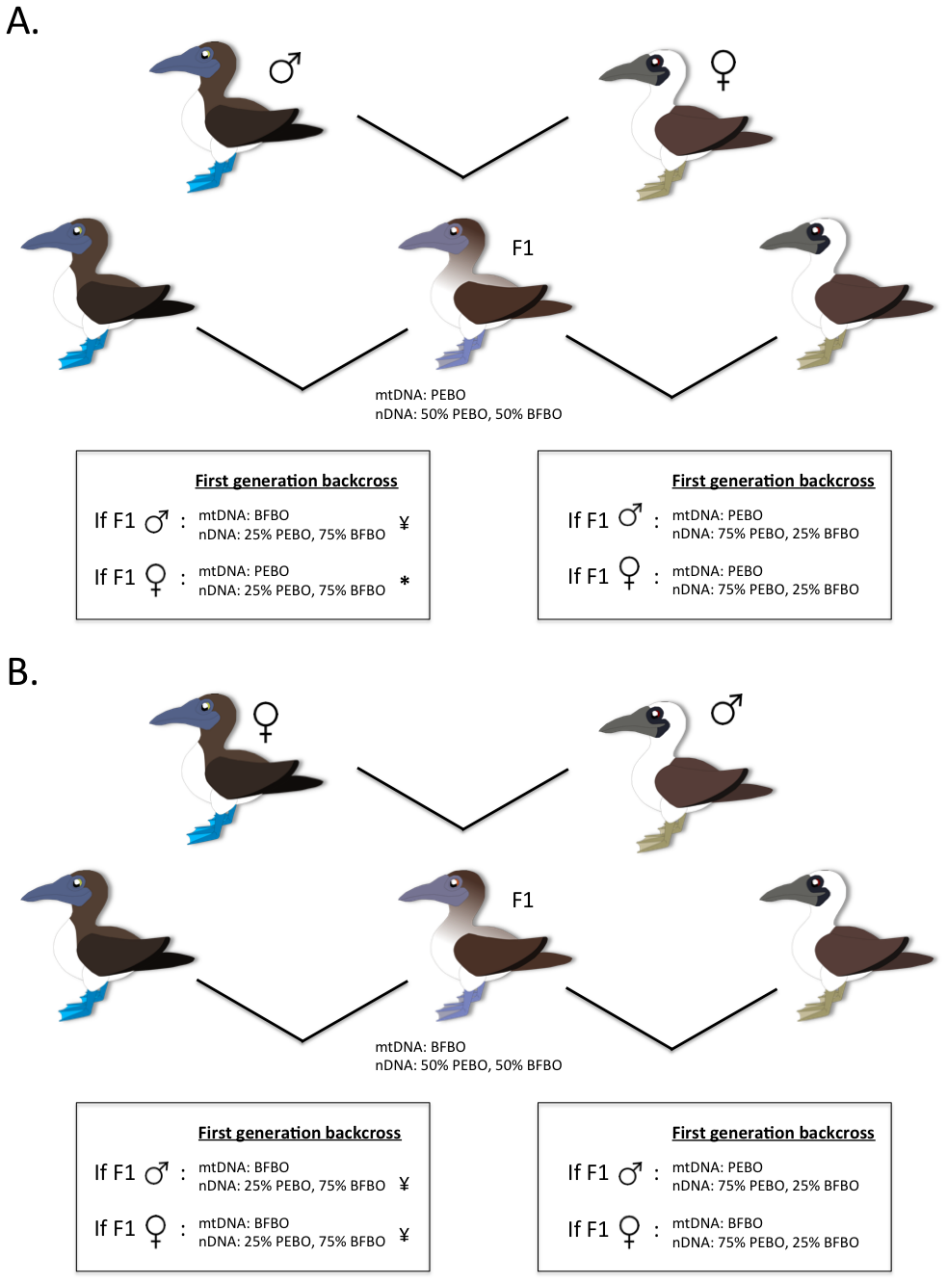
Theoretical crosses between blue-footed and Peruvian boobies and resulting mitochondrial and nuclear DNA complements in F1 and first generation backcross offspring. * indicates backcross observed by S.A. Taylor on Lobos de Afuera, ¥ indicates backcrosses that would lead to nuclear introgression of Peruvian booby DNA into blue-footed boobies without mitochondrial introgression. A) Hybrid cross observed in nature (all F1 individuals examined in Taylor et al (2012) possessed Peruvian booby mitochondrial DNA). B) Hybrid cross not yet observed in nature.

The asymmetrical gene exchange we detected, from Peruvian to blue-footed boobies, is generally consistent with recorded contemporary hybridization (Figure 4A)[32], [33], [34], [35]. All contemporary hybrids reported in Taylor et al. (2012) possessed Peruvian booby mitochondrial DNA and the nuclear genotype of F1 hybrids, suggesting they were the product of hybridization between a female Peruvian booby and a male blue-footed booby (Figure 4A). Furthermore, the majority were male. The single observed backcross event recorded by Taylor et al. (2012) occurred between an F1 female with Peruvian booby mtDNA and a male blue-footed booby, leading the authors to hypothesize that the majority of backcrossing of F1 individuals likely takes place between F1 females and male blue-footed boobies (Figure 4A). Though this backcross has been documented in nature [34], [35], it is likely less common than a cross between a male F1 and a female blue-footed booby, given that male F1 hybrids are more common than female F1 hybrids [34], [35]. Interestingly, successful and repeated backcrosses between male F1 hybrids and female blue-footed boobies would result in the pattern of asymmetrical gene exchange we detected using IMa.

A scenario where a female blue-footed booby chooses an F1 hybrid as a mate seems unlikely given the strong role of sexual selection in blue-footed booby mate choice [39], and the intermediate phenotype of F1 individuals [34]. That being said, the gene exchange between blue-footed and Peruvian boobies that we detected may have taken place before prezygotic barriers to gene exchange were as strong as they appear to be presently [35]. Although no studies have examined potential roles of foot colour in sexual selection for Peruvian boobies, adult morphology differs significantly [34] and these differences may lead to strong assortative mating [35]. It is important to note that Figure 4B illustrates a hybridization scenario that has not yet been documented in nature, and one that would be unexpected for the same reasons as a situation where a female blue-footed booby chooses an F1 hybrid as a mate, but that could result in the type of interspecific gene flow we report here. The possibility exists that this hybridization scenario occurred during the early stages of divergence between these species (Figure 4B).

### Ongoing or intermittent gene flow?

The strict allopatric model of divergence was rejected for dataset 1: the simplest model that fit our data included asymmetrical gene flow from Peruvian to blue-footed boobies (Table 3). Determining if gene flow was ongoing during the divergence process or if gene flow occurred between periods of allopatry, however, is impossible using the present data [8], [11], [13]. Although the contemporary ranges of the species may not reflect historical ranges, fossil evidence indicates that sulids have been present on the Humboldt Current Upwelling system and on islands in the North Equatorial Counter Current since the middle Miocene [40].

A number of aspects of the eastern tropical Pacific may have facilitated either gene flow between periods of alloparty, or regular gene flow between populations throughout divergence. Suitable breeding sites for large tropical seabirds are distributed in a stepping-stone pattern throughout the eastern tropical Pacific, which may have facilitated movement of individuals across large distances. As well, the marine environments in which blue-footed and Peruvian boobies forage were established prior to the divergence of the species [30], [31], [42] and, as it does for contemporary populations [28], [29], [35], this may have allowed relatively free movement of individuals across large distances. Finally, blue-footed and Peruvian boobies have the ability to travel long distances when food shortages occur, and have been recorded thousands of kilometres north and south of their contemporary ranges [43], [44]. All of these factors may have facilitated interspecific interaction, and gene flow, throughout the divergence process.

Given that IMa accounts for recent shared ancestry and uses a coalescent approach with multiple unlinked loci, it is unlikely that speciation between blue-footed and Peruvian boobies occurred in allopatry and that the gene flow we report is the result of recent secondary hybridization. The direction of gene flow is not what we would expect (backcrossing between male F1 hybrids and female blue-footed boobies) knowing that assortative mating is strong in contemporary colonies [35]. Potentially, the direction of gene flow we report from IMa analyses is further evidence that gene flow occurred at some point during the divergence process rather than following speciation in isolation: prior to the development of strong prezygotic isolation F1 male hybrids may have had more reproductive success than reported in Taylor et al. (2012).

### Summary

In the present paper we sought to evaluate the hypothesis that blue-footed and Peruvian boobies diverged from their common ancestor in a manner other than the classic allopatric model (i.e., with gene flow) using multiple autosomal, sex-linked, and mitochondrial loci, and to examine the role of the mitochondria and z chromosome in avian speciation. Findings indicate that asymmetrical gene flow occurred between Peruvian and blue-footed boobies during the divergence process, potentially between periods of allopatry. These results add to a growing body of literature highlighting that the classic allopatric model, divergence in the absence of gene flow, may not be as pervasive as previously thought.

## Supporting Information

Table S1 Summary of variation of introns screened in an initial test panel of blue-footed (*Sula nebouxii*) and Peruvian (*S. variegata*) boobies. Amp  =  amplification success, *N*  =  number of variable sites, *n*  =  number of individuals screened for variation. Loci used in the current study are highlighted in grey. ^†^Friesen *et al*., 1997, ^ψ^Friesen *et al*., 1999, ^‡^Backström et al. 2006, ^φ^Driskell & Christidis 2004, * V.L.F unpulb. data.
(DOCX)

Appendix S1 Phased haplotype sequences from alpha enolase and lipoprotein lipase. Sequences are less than 200 base pairs and cannot be submitted to GenBank.
(DOCX)
