# Supplementary material for: Evidence for Asymmetrical Divergence-Gene Flow of Nuclear Loci, but Not Mitochondrial Loci, between Seabird Sister Species: Blue-Footed (Sula nebouxii) and Peruvian (S. variegata) Boobies
Source: PLoS One. 2013 Apr 17;8(4):e62256. doi: 10.1371/journal.pone.0062256 (PMC3629132; doi:10.1371/journal.pone.0062256)
Supplement: Table S1 — Summary of variation of introns screened in an initial test panel of blue-footed (Sula nebouxii) and Peruvian (S. variegata) boobies. Amp = amplification success, N = number of variable sites, n = number of individuals screened for variation. Loci used in the current study are highlighted in grey. †Friesen et al., 1997, ψFriesen et al., 1999, ‡Backström et al. 2006, φDriskell & Christidis 2004, * V.L.F unpulb. data. (DOCX) [file pone.0062256.s001.docx]

**Table S1.**  Summary of variation of introns screened in an initial test panel of blue-footed (*Sula nebouxii*) and Peruvian (*S. variegata*) boobies. Amp = amplification success, *N* = number of variable sites, *n* = number of individuals screened for variation. Loci used in the current study are highlighted in grey. ^†^ Friesen *et al.,* 1997, ^ψ^ Friesen *et al.,* 1999, ^‡^ Backström et al. 2006, ^φ^Driskell & Christidis 2004, * V.L.F unpulb. data.

| **Intron** | **Amp** | **Location** | ***N* / *n*** | **Mutation rate (μ)** substitutions/locus/year |
| --- | --- | --- | --- | --- |
| IPO11^‡^ | N | Z chromosome | n/a |  |
| PARP8^‡^ | N | Z chromosome | n/a |  |
| 24105^‡^ | Y | Z chromosome | 2 / 5 | 0.00000014 |
| GAF^‡^ | Y | Z chromosome | 0 / 5 |  |
| ALDOB^‡^ | Y | Z chromosome | 2 / 5 | 0.00000028 |
| 25189^‡^ | Y | Z chromosome | 0 / 5 | 0.00000017 |
| CHD12 | Y | Z chromosome | 0 / 5 |  |
| ENOL^†^ | Y | Autosomal | 9 / 4 | 0.00000034 |
| CBA | Y | Autosomal | 0 / 6 |  |
| Crystalin* | Y | Autosomal | 0 / 6 |  |
| FOL* | Y | Autosomal | 2 / 7 |  |
| GPD^†^ | Y | Autosomal | 0 / 4 |  |
| LIPO* | Y | Autosomal | 1 / 9 | 0.00000029 |
| OD ^ψ^ | Y | Autosomal | 1 / 8 |  |
| LAM^†^ | Y | Autosomal | 0 / 4 |  |
| Rhodopsin 1* | Y | Autosomal | 0 / 4 |  |
| RP40 ^ψ^ | Y | Autosomal | 0 / 8 |  |
| TIM* | Y | Autosomal | 2 / 10 | 0.00000036 |
| FIB^φ^ | Y | Autosomal | 1 / 10 | 0.00000065 |
