# Supplementary material for: Evidence for Asymmetrical Divergence-Gene Flow of Nuclear Loci, but Not Mitochondrial Loci, between Seabird Sister Species: Blue-Footed (Sula nebouxii) and Peruvian (S. variegata) Boobies
Source: PLoS One. 2013 Apr 17;8(4):e62256. doi: 10.1371/journal.pone.0062256 (PMC3629132; doi:10.1371/journal.pone.0062256)
Supplement: Appendix S1 — Phased haplotype sequences from alpha enolase and lipoprotein lipase. Sequences are less than 200 base pairs and cannot be submitted to GenBank. (DOCX) [file pone.0062256.s002.docx]

**Appendix 1**

**Alpha enolase intron sequence:**

**LT259-a CTGTAAGAGGGGTCAAGAGCAGAACAAACAGCTAGCCTTTTACAGGGATCTTGGGAATATTTTTTTGAGGTGGGTAGTACTTGGCTGCAGCACTTTGGTACCACTGCTTTCAAACAAGACCTTTCAGGGTGGCGTTGTCTTAATGCTCCTTGTATCCCTCTACAG**

**LP-53-b CTGTAAGAGGGGTCAAGAGCAGAACAAACAGCTAGCCTTTTACAGGGATCTTGGGAATATTTTTTTGAGGTGGGTAGTACTTGGCTGCAGCTCTTTGGTACCACTGCTTTCAAACAAGACCTTTCAGGGTGGCGTTGTCTTAATGCTCCTTGTATCCCTCTACAG**

**T03-b CTGTAAGAGGGGTCAAGAGCAGAACAAACAGCTAGCCTTTTACAGGGATCTTGGGAATATTTTTTTGAGGTGGGTAGTACTTGGCTGCAGCACTTTGGTACCACTGCTTTCAAACAAGACCCTTCAGAGTGGCGTTGTCTTAATGCTCCTTGTATCCCTCTACAG**

**T10-b CTGTAAGAGGGGTCAAGAGCAGAACAAACAGCTAGCCTTTTACAGGGATCTTGGGAATATTTTTTTGAGGTGGGTAGTACTTGGCTGCAGCACTTTGGTACCACTGCTTTCAAACAAGACCTTTCAGAGTGGCGTTGTCTTAATGCTCCTTGTATCCCTCTACAG**

**IP21-b CTGTAAGAGGGGTCAAGAGCAGAACAAACAGCTAGCCTTTTACAGGGATCTTGGGAATATTTTTTTGAGGTGGGTAGTACTTGGCTGCAGCACTTTGGTACCACTGCTTTCAAACAAGACCTTTCAGGGTGGTGTTGTCTTAATGCTCCTTGTATCCCTCTACAG**

**Lipoprotein lipase intron sequence:**

**C004-a TTTTACCACACTCCTGATAAGCCGTGCTAGATACGTCAGCTGAGGTATCTATACACTGCAGATAATATTAAGATATCTCCTTTTTTAATTGAAAAAAACCTCAACAGGTGACAG**

**ER12-a**

**TTTTACCACACTCCTGATAAGCCGTGCTAGATACGTCAGCTGAGGTATCTATAGACTGCAGATAATATTAAGATATCTCCTTTTTTAATTGAAAAAAACCTCAACAGGTGACAG**
